# Intramedullary erythrophagocytosis in myelodysplastic syndrome with heterozygous U2AF1 Q157R variant

**DOI:** 10.1002/jha2.1084

**Published:** 2025-02-07

**Authors:** Kritika Krishnamurthy, Aditi Shastri, Yanhua Wang

**Affiliations:** ^1^ Montefiore Medical Center Bronx New York USA; ^2^ Albert Einstein College of Medicine Bronx New York USA

**Keywords:** bone marrow histology, erythrophagocytosis, hemolysis, myelodysplastic syndrome, U2AF1

## Abstract

This report highlights a somewhat unique case of U2AF1 mutated myelodysplastic syndrome (MDS) with morphological evidence of increased intramedullary erythrophagocytosis, in the absence of obvious clinical signs of hemolysis. These findings merit investigation in a larger cohort of U2AF1 mutated MDS cases to further delineate the morphological spectrum of ineffective intramedullary hematopoiesis and nonimmune hemolysis, including features distinctive to S34 and Q157 variants.

1

Sir: A 65‐year‐old Hispanic man with no significant past medical history presented to the emergency department from his primary care physician for anemia and thrombocytopenia and was incidentally found to have a pulmonary embolus for which he was started on Lovenox. His blood work revealed the following red cell indices: hemoglobin 6.51 mmol/L, RBC count 3.44 × 10^12^/L, hematocrit 34.3, mean corpuscular volume 99.7 fl, mean corpuscular hemoglobin of 30.5 pg, and red cell distribution width 19.3%. The absolute retic count was 147.30 × 10^9^/L (4.3%) with 26% immature reticulocyte fractions. The serum lactate dehydrogenase was mildly elevated at 271 IU/dL. A direct Coombs test was negative. There were no clinical signs or evidence of hemolysis. The platelet count was 65 × 10^12^/L, while the WBC count was 10.5 × 10^9^/L, with 2% blasts on the peripheral smear. Flow cytometry on the peripheral blood showed 8% CD34 and CD117 positive blasts with decreased CD13 expression and dim to negative CD38 suspicious for a myeloid neoplasm.

A bone marrow biopsy with aspirate was performed and microscopic evaluation showed a hypercellular marrow (90%) with left‐shifted myeloid hyperplasia and 5% myeloblasts as highlighted by CD34 and CD117 immunohistochemical stains. The myeloid to erythroid ratio was increased and megakaryocytes were reduced in number with some small, hypolobated forms. There was a striking increase in the number of macrophages, the majority of which had maturing red cells and erythroid precursors engulfed in the cytoplasm. Cytogenetic analysis of three unstimulated and 17 stimulated bone marrow cells revealed deletion of the long arm of chromosome 7 at the band (q21) in 10% of cells. Next‐generation sequencing performed on the bone marrow aspirate revealed pathogenic frameshift variants in ASXL1 (VAF 27%) and ETV6 (VAF 47.5%), nonsense mutations in CREBBP (VAF 49.2%) and RAD21 (VAF 16.7%), and missense variant in U2AF1 (VAF 50.1%), in addition to several variants of unknown clinical significance in ANKRD26, CBLC, EPHA7, FANCF, FLT3, IL7R, KMT2C, NOTCH3, PIK3CA, and WT1.

Based on the above findings, a diagnosis of myelodysplastic syndrome (MDS), IPSS‐M high risk, was established, and the patient was started on metabolically optimized low‐dose dacogen and venetoclax trial. Subsequent bone marrow biopsy, performed 6 weeks post‐treatment onset, showed similar findings as the previous specimen in terms of hypercellularity and left‐shifted myeloid hyperplasia; however, the blasts percentage had decreased to 1.13% as seen on flow cytometry of the aspirate. The macrophages with engulfed red cells and erythroid precursors were still present in significant numbers.

Hemolysis is well‐documented in a subset of MDS cases. However, the pathobiological basis remains ambiguous, though many reports have suggested a nonimmune etiology. The possibilities include ineffective intramedullary erythropoiesis, acquired hemoglobinopathies, RBC membrane defects, red cell enzymopathy, or altered metabolic properties, as a result of aberrant/altered gene expression or product and subsequent effect on downstream signaling. The case presented above provides the first reported morphological evidence of the perturbed intramedullary hematopoiesis with increased erythroid lineage destruction by erythrophagocytosis of maturing red cells and erythroid precursors. The patient, however, did not have any other clinical features of hemolysis except an elevated reticulocyte count.

In a recent study by Komrokji et al. [1], U2AF1 mutations, in addition to being associated with worse outcomes, were observed in 30% of patients with hemolysis and occurred almost exclusively at the S34 hotspot. U2AF1 (U2 Small Nuclear RNA Auxiliary Factor 1) belongs to the splicing modulator family of genes and its gene product is critical for RNA splicing [2, 3]. Heterozygous mutations in U2AF1 have been reported in MDS in about 11% of patients and exclusively involve the S34 or Q157 amino acid residues that are located within the zinc finger motifs [2, 3, 4]. The S34 hotspot mutations have been shown to lead to differentiation defects in hematopoietic stem cells leading to ineffective hematopoiesis in experimental murine models [2]. Q157 mutations are more specifically associated with anemia in MDS. Zhang et al. [5] demonstrated the critical role of U2AF1 in erythropoiesis, including the considerable expression of the gene product in progenitor burst‐forming‐unit and colony‐forming‐unit stages of erythroid maturation. Zhu et al. [6] showed *U2AF1*‐mutations facilitated an autophagy flux via FOXO3a‐dependent apoptosis and NLRP3 inflammasome activation, which induced caspase‐1‐dependent programmed cell death, particularly pyroptosis. Pyroptosis is lytic in nature and results in the release of damage‐associated molecular patterns, and subsequent priming and recruitment of phagocytes [7].

In addition, an isolated 7q21 deletion was seen in 10% of cells in the current case. Cytogenetic abnormalities such as 5q deletion and 20q deletion have been reported in association with structural defects in RBCs such as elliptocytosis, spherocytosis, and poikiloanisocytosis which may contribute to the hemolysis. However, no such association between chromosome 7 deletion and RBC structural defects has been reported so far.

In conclusion, this report highlights a somewhat unique case of U2AF1 mutated MDS with morphological evidence of increased intramedullary erythrophagocytosis, in the absence of obvious clinical signs of hemolysis. These findings merit investigation in a larger cohort of U2AF1 mutated MDS cases to further delineate the morphological spectrum of ineffective intramedullary hematopoiesis and nonimmune hemolysis, including features distinctive to S34 and Q157 variants (Figures 1 and 2).

**FIGURE 1:**
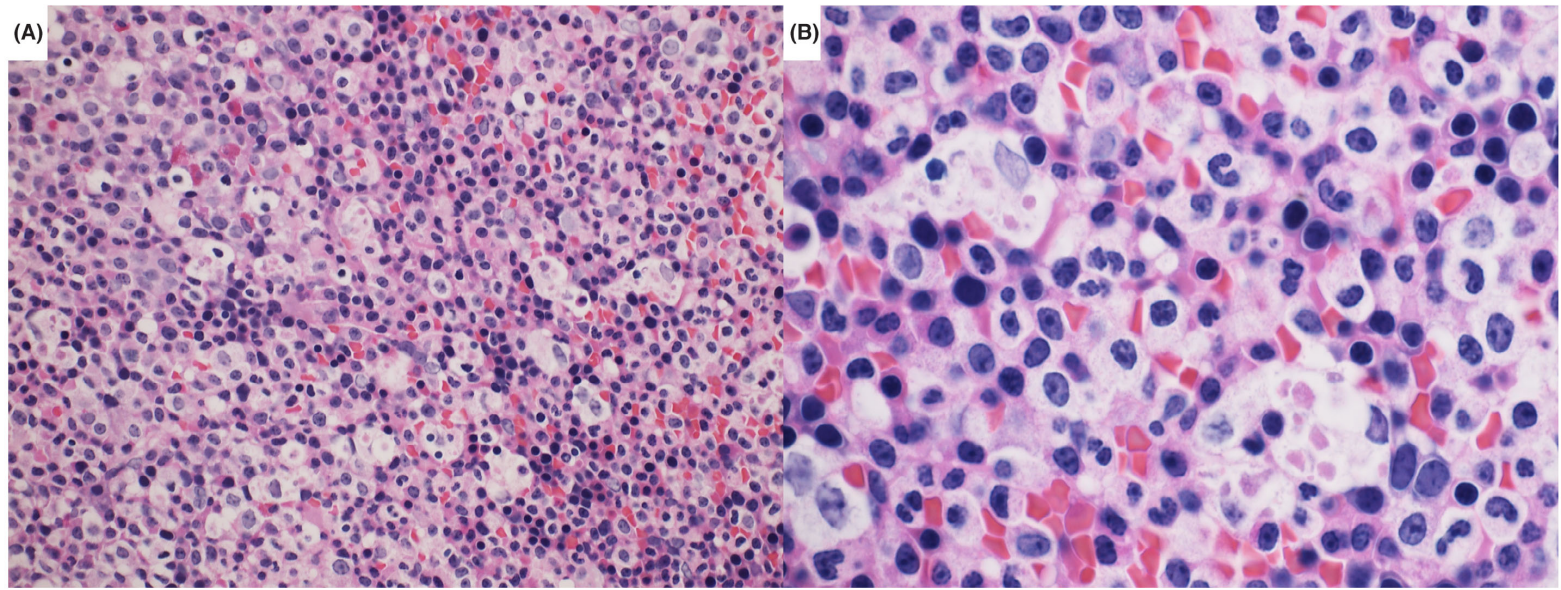
Hematoxylin and eosin‐stained bone marrow biopsy. (A) Low‐power view showing hypercellular marrow with left‐shifted myeloid hyperplasia with increased macrophages phagocytosing cellular debris. (B) High‐power view showing maturing red cells and erythroid precursors engulfed by macrophages in the marrow.

**FIGURE 2:**
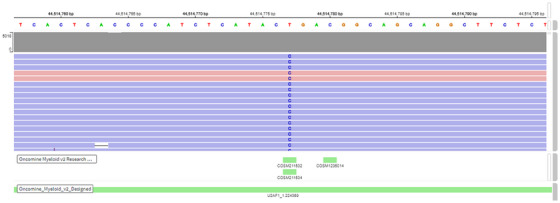
Integrated genomic viewer showing the U2AF1 Q157 variant identified by next‐generation sequencing.

## AUTHOR CONTRIBUTIONS

KK: Conceptualization; data curation; formal analysis; funding acquisition; investigation; methodology; project administration; resources; software; supervision; validation; visualization; roles/writing—original draft; and writing—review and editing. AS: Conceptualization; data curation; visualization; and writing—review and editing. YW: Conceptualization; funding acquisition; methodology; project administration; resources; supervision; visualization; and writing—review and editing.

## CONFLICT OF INTEREST STATEMENT

The authors declare that they have no conflict of interest.

## FUNDING INFORMATION

NA

## ETHICS APPROVAL STATEMENT

The authors have confirmed ethical approval statement is not needed for this submission.

## PATIENT CONSENT STATEMENT

The authors have confirmed patient consent statement is not needed for this submission.

## CLINICAL TRIAL REGISTRATION (INCLUDING TRIAL NUMBER)

The authors have confirmed clinical trial registration is not needed for this submission.

## Data Availability

NA

## References

[1] Komrokji R , Aguirre LE , Al Ali N , Hussaini M , Sallman D , Rollison D , et al. U2AF1 and EZH2 mutations are associated with nonimmune hemolytic anemia in myelodysplastic syndromes. Blood Adv. 2023;7(1):1–8. 36129843 10.1182/bloodadvances.2022007504 PMC9813529

[2] Shirai CL , Ley JN , White BS , Kim S , Tibbitts J , Shao J , et al. Mutant U2AF1 expression alters hematopoiesis and pre‐mRNA splicing in vivo. Cancer Cell. 2015;27(5):631–643. 25965570 10.1016/j.ccell.2015.04.008 PMC4430854

[3] Zamore PD , Green MR . Identification, purification, and biochemical characterization of U2 small nuclear ribonucleoprotein auxiliary factor. Proc Natl Acad Sci U S A. 1989;86(23):9243–9247. 2531895 10.1073/pnas.86.23.9243 PMC298470

[4] Nian Q , Li Y , Li J , Zhao L , Rodrigues Lima F , Zeng J , et al. U2AF1 in various neoplastic diseases and relevant targeted therapies for malignant cancers with complex mutations (Review). Oncol Rep. 2024;51(1):5. 37975232 10.3892/or.2023.8664 PMC10688450

[5] Zhang J , Zhao H , Wu K , Peng Y , Han X , Zhang H , et al. Knockdown of spliceosome U2AF1 significantly inhibits the development of human erythroid cells. J Cell Mol Med. 2019;23(8):5076–5086. 31144421 10.1111/jcmm.14370 PMC6652819

[6] Zhu Y , Song D , Guo J , Jin J , Tao Y , Zhang Z , et al. U2AF1 mutation promotes tumorigenicity through facilitating autophagy flux mediated by FOXO3a activation in myelodysplastic syndromes. Cell Death Dis. 2021;12(7):655. 34183647 10.1038/s41419-021-03573-3 PMC8238956

[7] Kolb JP , 3rd Oguin TH , Oberst A , Martinez J . Programmed cell death and inflammation: winter is coming. Trends Immunol. 2017;38(10):705–718. 28734635 10.1016/j.it.2017.06.009 PMC5710799

